# A Practical Treatise on Urethritis and Syphilis

**Published:** 1836-10-01

**Authors:** 


### A PRACTICAL TREATISE ON URETHRITIS AND SYPHILIS, &c. &c. By *William Henry Judd,* Surgeon to His Majesty's Fusileer Guards, &c. &c.

[Second and concluding Article.]

IN the 48th number of this Journal—that for April of the present year—we noticed at some length the first part of the volume which is now before us, and dispatched the consideration of primary venereal symptoms. We promised, at the conclusion of that article, to return in the succeeding number to the subject of lues, and to lay before our readers an ample notice of that portion of the work of Mr. Judd which treats of its secondary consequences. Accident prevented our redeeming that promise in our last number, and we therefore return to the task in this.

If such of our readers as take an interest, and all should do so, in the treatment of the venereal disorder, will revert to our notice of the first part of Mr. Judd's work, they will find that we took exception to many of his inferences, and offered some reasons for doubting whether he had thrown much light on the primary features of this singular malady. With minds perfectly unbiassed in any way, we proceed to examine our author's conclusions with respect to its secondary symptoms, and if we have a leaning, it is towards partiality and praise. We know Mr. Judd to be an unaffected, industrious, and honourable man, and it may be readily supposed that we can feel no inclination to point against him the severity of criticism. But placed as we are, there is one consideration paramount to all others—the necessity of discharging our duty to the public, and of offering an honest, though probably a far from able, estimate of the work that we take upon us the responsibility of reviewing.

Two hundred and twenty pages were traversed in our last notice. They were passed with speed, for the great majority were occupied with cases, which, proving little save the industry of the observer, were easily disposed of.

Three hundred and forty-eight remain, and we are not quite sure that they will admit of absolute and beneficial compression into the sixteen or twenty that our engagements enable us to afford them. For not only are those pages occupied with a view of venereal secondary symptoms, but a chapter is devoted to the hydriodate of potass, a remedy which is now extensively used, will probably be much abused, and therefore demands from us more than usual attention. Probably we shall throw our observations on this head into the shape of a separate article. We shall see about this bye-and-bye.

### Venereal Eruptions.

It has been doubted by those who either have no such organs as eyes, or believers, perhaps, in the doctrine of innate ideas, have philosophically determined not to use them, whether such things as venereal eruptions exist. Persons of ordinary intellect will probably be more convinced that they do, from actually seeing their occurrence, than from reading the best metaphysical proof that the subtlest disputant could offer. But Mr. Judd starts with some analogical considerations, which he thinks calculated to elucidate the question.

Were it needful, he remarks, to prove, for the satisfaction of the scientific, that venereal poisons produce roseola, ecthyma, and so on, it would only be necessary to point out to them the similar eruptions that follow the application of morbid poisons to the surface of the body, or to its interior. If copaiba, for example, swallowed produce a rash upon the skin, is not that evidence, argues Mr. Judd, that lues does the same? He carries this analogical argument to some extent, and on mixing up with it portions of other analogies also, there results the following passage.

"Now enough has been brought to mind, to show the reader that roseola is often both the produce of unwholesome chyme, and also of animal poisons from muscles, pus, etc.; and why should not venereal roseola be the produce of that particular poison? and occupy portions of cuticle in the form of puniceous patch, etc., as after other poisons? or, when more intense, why should it not rise into papulæ, like erythema papulatum? Though, I confess, from our too limited knowledge of the relations existing between cause and effect, I know not why venereal mottled skin is at times but a delicate exanthematous rash over the whole body, and at others grouped into strongly-marked puniceous patches—or why, in instances, it is bounded into circular ones, as in maculæ coccineæ. But the facts and appearances are such, that we might reasonably infer by analogy, that Nature had formed these strong resemblances as a link of connexion, to bind together what appear to be the most distant with the most intimate forms of eruption, and yet without destroying their beauty, symmetry and variety, by jumbling them together as one cutaneous affection. It is quite curious to observe how much eruptions occasioned by copaiba (probably from causing the chyme to be poisonous to the system) resemble the puniceous patches occasioned by venereal virus, as also those produced by urethritis: indeed they cannot be said to differ, except in the more diminutive size of the patches of the former. If I wished to define Nature's method of producing the puniceous patch of mottled skin, the papulæ of lichen, the vesicle of rupia, or the pustulæ of ecthyma, I would take as a simile the blushes arising by simple friction upon the skin, and compare them with the irritation produced by animal poisons: and what, let me ask, would be the effect of the farmer's labourer lacing his boot too tight? or the soldier blistering his foot by a march; why a small degree of friction from either cause would induce redness or erythema; and if carried a degree or two further, it would, by inflammatory action, occasion lymph to be thrown out, and generate a blister or vesicle; and a degree of friction a little beyond this would lead to the formation of pus, and make the latter a pustule: and thus all the intermediate stages will have been gone through, from the erythema or puniceous patch, to the throwing out of clear lymph, as in the vesicle; or to the secretion, so produced, becoming opaque lymph, or genuine pus, as in ecthyma: these are but similar changes to those we observe in the vaccine vesicle, or in a vesicle that becomes a pustule by the mere friction of the clothes. After taking these few simple and familiar examples into consideration, surely no one will say, that the changes in eruptions from mere cuticular redness to enlarged papillæ and tubercles, or even from vesicles to pustules, are so wide, or so great, that they surpass our comprehension! Nor will any one be surprised at the curious, and almost endless variety of papulæ, vesiculæ, and pustulæ, so frequently exhibited during an almost continued eruption; or that one form should be changed into another, like a metamorphosis in Ovid!" 225.

We confess that, were we sceptical before, our doubts would not be materially dispelled by the preceding argument. Like a skilful painter, the author has thrown into it a dash of the *chiaro-scuro*, which may heighten the effect, but diminishes, in some degree, the clearness. The rigid reasoner will probably observe, that the passage before us appears to embody two analogies essentially distinct:—The first, that as various substances, applied to the interior or exterior of the body, occasion eruptions, secondary in point of occurrence, on the latter, so does, or rather may, the venereal poison;—the second, that as local irritants of various intensities produce various degrees of irritation, so are the various venereal eruptions to be regarded as evidence of various amounts of primary venereal virus.

The first analogy is sufficiently correct, and we can perceive little difference, in principle, between the eruption of variola, produced by the inoculation of the primary poison, and the eruptive sequelæ of lues, presented by the inoculation of it. The analogy, we say, is a good one; but, in point of fact, it is unnecessary, because the evidence is just as strong on one side as on the other, and we only rob Peter to pay Paul, if we attach more relative credit to either.

But the second analogy is demonstrably fallacious. If a man chafes his heel by a walk with a tight boot on one day, and produces erythema—and if, by walking a second day with the same boot, he frets the irritated surface to a blister, the local irritant is obviously increased in intensity, and the local irritation is proportionally augmented. But if the same individual, from one infection, has first lichen and then pustule, the sequelæ differ indeed, but what evidence have we of the primary irritant being augmented. The analogy is not illustrative, for the similarity of the premises, necessary to constitute the analogy, is assumed. A pregnant instance this of the unfortunate laxity of reasoning that prevails in medical works—a laxity that accounts satisfactorily enough for the opposite conclusions of medical inquirers on all medical subjects.

On these and some similar arguments, Mr. Judd grounds the following table of—

"*The Seven Orders of Venereal Eruptions.*

ORDER I.
EXANTHEMATA.

1. EXANTHEMA ROSEOLUM.
2. ——— PUNICEUM.

ORDER II.
PAPULÆ.

1. PAPULÆ ELONGATÆ.

2. LICHEN EXANTHEMATICUS.

3. ——— RACEMOSUS.

4. ——— SOLITARIUS.

5. ——— CIRCUMSCRIPTUS.

6. ——— VESICULARIS.

ORDER III.
VESICULÆ.

1. HERPES SOLITARIUS.
2. ——— CONFERTUS.
3. ——— CIRCINATUS.
4. RUPIA SIMPLEX.
5. ——— PROMINENS.

ORDER IV.
PUSTULÆ.

1. ECTHYMA PHLYZACIUM.
2. ——— PSYDRACIUM.
3. ——— MAGNUM.

ORDER V.

MACULÆ.

1. SPILI COCCINEI.
2. ——— CRUENTATI.
3. ——— CUPREI.

ORDER VI.
TUBERCULA.

1. PHYMATOSIS OVATA.
2. ——— ANNULATA.
3. ——— VERRUCOSA.*

ORDER VII.
SQUAMÆ.

1. LEPRA VENEREA.
2. PSORIASIS.
3. ——— PALMARIA."

We see no material objections to this classification of venereal secondary eruptions. Yet it does not comprise all their known forms.

1. In peculiar constitutions, secondary eruptions of a vesicular and pustular character follow venereal infection, and those eruptions admit of arrangement, either under the heads of rupia or ecthyma. But not unfrequently ulcerations occur on the integument, which cannot fairly be termed ei-

* "The word VENEREUS may be added after each genus, to distinguish them from others in the arrangement of Cullen, etc."

ther rupia or ecthyma at their commencement, and differ materially from them in their progress. The ala nasi, for example, in an unfortunate gentleman now under our care, became tumid, red, and painful. A minute spot of ulceration formed on the swollen part. The ulcer deepened and spread. When it had nearly destroyed the ala, it began to heal on one side, whilst it extended on the other, and thus a creeping foul ulceration was established. A similar sore formed on each leg, though, in these instances, the ulceration commenced with a vesicle, the fluid in which became turbid and partially dried. We mention the case as an illustration only of what not unfrequently occurs. Creeping or serpiginous ulcerations, of a phagedænic character, may constitute distinct sequelæ of venereal sores, and, as they cannot be justly considered either pure rupia or ecthyma, they deserve a niche by themselves.

M. Rayer, who offers a very tolerable account of primary and secondary syphilis, admits, in his enumeration of the latter, "secondary cutaneous ulcers."

2. There is a form of tubercle of which, so far as we can perceive, no notice is taken by our author. It is the subcutaneous tubercle. No doubt this is usually a cachectic, that is, a mixed symptom ; but we have seen it occur where mercury had not been taken, and where it had all the characters of a pure secondary affection.

3. Baldness from syphilis, and alterations of the nails, which may be considered strictly as cutaneous alterations, are not included in the present classification. Neither is common—the former, indeed, uncomplicated with venereal eruptions, is rare. Yet they do occur, and they should not, therefore, be totally excluded from the pages of a treatise on the venereal disease.

Such appear to us to be the principal omissions in Mr. Judd's table of the seven orders of venereal eruptions. Candour must admit that they are not very serious. Perhaps it may be doubted if he has not unnecessarily multiplied the varieties of the particular kinds of eruption. That lichen, for example, takes on the exanthematic, racemose, solitary, circumscribed, and vesicular characters, is true ; but it is probable that minute and ingenious observation would distinguish many more, and the tendency, of late, has been to swamp these variable and indefinite distinctions between the same eruption in different cases, or at different times—distinctions which often perplex rather than instruct, and insensibly, perhaps, but frequently, lead the mind from the consideration of general and important, to minute and trivial particulars. We leave this, however to the taste of the individual practitioner, and, possibly, it is better to be too minute than too superficial.

If the inexperienced surgeon casts a glance on such a classification as the one before us, he perceives how numerous, varied, and different syphilitic eruptions are. They occupy the majority of the orders, in which cutaneous diseases in general can be included, and they form a study whose difficulty is fully proportioned to its interest. If he reflects that these eruptions form but a portion of venereal symptoms—that the alterations of the throat are nearly as numerous as the eruptions, and the diseases of the fibrous and osseous tissues as complicated as either, and more serious—he will ask himself, if possessed of intelligence and candour—" Is it right that I should devote no more time to the investigation of this malady, than I see my fellow-students do? is it justifiable that, seeing little, reading nothing, of a complaint so mysterious and so severe, a complaint which, I perceive, forms a study in itself, I should go into practice, determined to treat what I am not by any means acquainted with, and prepared to exhibit blindly, in a dangerous disorder, an equally dangerous remedy?" Such is the question that many a young practitioner must have asked himself. What has been the answer? Let London consultations—broken constitutions—and mutilated bodies tell. We return to Mr. Judd.*

### I. Venereal Exanthemata.

"Rashes, of various extent and intensity, formed in patches, differently figured, and of every shade from delicate pink to rose red, or deep crimson, at times a little risen, and now and then accompanied by fever and redness of the fauces; various in duration, and in most instances followed by slight cutaneous desquamation, but not to the extent usual after scarlatina. Caused by a morbific poison circulating in the system, they generally appear about nine weeks after contamination, occur both with and without bubo, and are non-contagious." 228.

The order of exanthemata contains, as our readers will see by reference to the table of classification, two genera—1, exanthema roseolum; and, 2, exanthema puniceum.

#### 1. *Exanthema Roseolum.*

"Exanthema roseolum is a diffused efflorescence, or rash, preceded by slight fever and pains in the limbs, situated beneath the cuticle, formed by increased determination of blood to the vessels of the rete mucosum; and at times with slight extravasation, which, presenting its colour through the partially transparent cuticle, gives an elegant pink or rose colour, its intensity varying in different individuals, from the slightest perceptible tint, to the deepest roseate colour. It has no set pattern, and is irregular, and separated by larger portions of natural skin. The papillæ are not generally raised, as in scarlatina or rubeola; and, unlike other exanthemata, this eruption commences on the abdomen. That this appearance is the effect of an increased quantity of blood in the cutaneous vessels, and a slight extravasation of coloured serum, at times, is tolerably proved, by the circumstance of a little pressure driving the fluid from the red portions, and the skin immediately resuming its natural colour, and from the tint recurring in the part so soon as the pressure is removed." 229.

Mr. Judd judiciously observes, that this not unfrequently precedes other venereal eruptions, and is itself often anticipated by pains, especially, he thinks, in the legs, below the calf. It is usually, also, so far as we have seen, ushered in by symptoms of pyrexia, which occasionally are severe, and attended with inflammation of the thoracic, mucous, or serous membranes. The same is the case with lichen, and we quite agree with Mr. Judd in the opinion, that a strong relationship exists between this latter and the venereal

---

* We shall not enter fully on the subject of secondary eruptions at present, as we anticipate the occurrence of a better opportunity anon.

exanthematous eruptions, although Rayer, we observe, dissociates them altogether.

Mr. Judd relates a case in which a burrowing sore, in the fold of the prepuce, was succeeded by "exanthema roseolum," which itself was followed by papulæ, iritis, and, finally, by ecthyma. It is not necessary to pause for the details. We therefore proceed to the second genus, christened—

2. *Exanthema Puniceum, or Puniceous Patch.*

Mr. Judd uses this new term to designate a crimson-coloured venereal eruption, consisting of very numerous and distinct patches, of much more intense colour and set pattern than the roseoloid eruption. By carefully picking out from the midst of other passages the following, we may present a consistent description of this affection.

So far as Mr. J. can determine, the sores producing the "puniceous patch" arise as vesicles. But if we reflect how many venereal sores commence as such, and how many vesicles on the generative organs never become true venereal sores at all, we shall not feel disposed to attach much importance to this observation.

The puniceous patch seldom begins later than about nine weeks after absorption of the poison. Such is the statement of our author, but it must be evidently received cum grano salis, for how are we to determine in many cases when absorption actually takes place?

It is usually ushered in by febrile symptoms, and pains in the shoulders and hips, whilst it is itself the occasional precursor of other eruptions, as lichen, herpes, and so forth. It is frequently attended with redness of the fauces or sore throat. It is at times the effect of poisonous substances taken into the stomach, such as muscles, copaiba, &c. With something like inconsistency, Mr. Judd, after observing that it may follow the exhibition of copaiba, declares that it differs much in size, elevation, and character from any eruption that he finds described. Now the eruption from copaiba, poisonous shell fish, &c. is sufficiently familiar, and has been described under the name of urticaria febrilis—while the syphilitic exanthematous eruption is familiar also, and is commonly called syphilitic roseola. But to proceed. The puniceous patches are small, and seldom form a continuous eruption. The centre of each patch is raised, its form is usually an irregular lozenge or hexagon, and although the most raised patches may in their centre approach an oval, yet this is still set upon a lozenge-shaped base.

"On sliding the hand over the cuticle it detects a raised irregularity of surface, corresponding to the irregularity of the shapes of the patches, which are also perceptible to the eye, as being distinctly raised above its less elevated crimson base. Each patch both feels and looks as if lymph had been thrown out upon the face of the absorbent rete mucosum; and I am next to certain that this effusion is given out between the rows of what are called papillæ, from the following accidental experiment. Whilst modelling an eruption, I cut a slice of cuticle off the end of my ring-finger, and very accurately laid bare the cutis vera, when a watery serum slowly issued in minute drops upon the newly exposed surface. These I frequently dried off by touching them with bibulous paper, and saw, through a powerful lens, fresh drops issue from between the rows of papillæ in the cutis, and lodge in lines correspondent to the natural folds in the cuticle and cutis; and whenever their surfaces became overloaded to confluence, the minute drops of fluid invariably closed inwards from the circumference, and the red liquid formed lozenge-shaped patches on the rete mucosum, and only required the cuticular covering to complete their exact resemblance in shape and shade to puniceous patches. This squarish, or lozenge-like, form is much indulged in by nature, for the bundles of fibres composing many of the muscles are of this form, as may be seen in those of the round of beef, where a cross section of its flesh shews the fact on a large scale. And such is the true cause of the form of these red patches, their shape being in some measure determined by the deep sulci that divide the cuticle and its adjoining membrane into triangular, lozenge-shaped, and polygonal eminences." 239.

Mr. Judd, in his declaration that lozenge-like forms are greatly affected by nature, has perhaps in his recollection the opinion of the famous Sir Thomas Browne, that Nature rejoices much in quincunxes. Sir Thomas carries out this theory in his Garden of Cyrus, and Mr. Coleridge has pleasantly remarked that " he finds quincunxes in heaven above, quincunxes in earth below, quincunxes in the mind of man, quincunxes in tones, in optic nerves, in roots of trees, in leaves, in every thing." So we see that old Sir Thomas has anticipated Mr. Judd.

The puniceous patch runs a variable course in point of time. Its stains rarely last beyond four weeks, but it leaves on its decline " a feel like thickened, raised, patches of skin." As it is often ushered in by fever, it is not surprising if pains in the chest, hoarseness, inflammation, in short, of the mucous or the serous membrane should occasionally be developed. It sometimes ends, according to our author, in consumption or ascites. Both the pleurisy at the commencement, and the sequelæ we have mentioned, cannot be regarded as any thing more than accidental complications or results. Fever being set up, inflammatory affections will occur in particular habits.

Such is the description offered by our author of the " puniceous patch." Eight cases are related in illustration. In one it followed a vesicle upon the penis. In the second, it succeeded a round sore upon the penis and suppurating bubo. In the third, it was consecutive to a flat superficial sore, which commenced as a vesicle. In the fourth, it followed a " large excavated ugly sore," which left on healing a hard cicatrix, and a dull red stain. In the fifth, it followed a sloughy sore, and articular inflammation and effusion. In the sixth it followed " a suspicious-looking, sloughy little sore," surrounded by induration. It was succeeded by lichen. In the seventh, the primary symptoms were " an abrasion, and a tender red syphilitic wart." The abrasion became a foul sloughy sore, which actually sloughed and gave rise to much hæmorrhage. A bubo in the groin suppurated, ulcerated, and " sloughed frightfully," leaving " a very large chasm." Ecthyma appeared—the puniceous patch followed—and again ecthyma came out.

The eighth case may usefully receive a more particular notice.

### II. Papulæ.

These form the second order of our author's classification. He is extremely anxious to shew that there is not an abrupt interval between the exanthematous eruptions and papulæ. This is so natural and just a view that none can consistently object to it. Mr. Judd, however, appears to anticipate some cavilling, and he points out the gradual manner, in which the two eruptions run into each other.

" Take," he says, "the delicate exanthismous skin; place next it the slightly marked puniceous patch; let that be followed by an instance of the more intense and higher raised variety of the same eruption, and lastly by puniceous patch set with enlarged papulæ, and we have then, from a flat surface, absolutely arrived gradatim at a miniature representation of clustered lichen, which is going a step beyond what we aimed at, viz. simple lichen. The gradations we have just traced is like to those of a traveller starting upon a plane, rising by a gentle ascent, and arriving at the summit of a mountain: and as if nature were more than ordinarily anxious to connect all the various shades and varieties in links, I have of late discovered (and I believe I am the first that has done so), that puniceous patches on the scrotum (a case of which kind has just been given) at times actually have minute papulæ set on them, and even, though less frequently, vesicles upon the lozenge-like puniceous bases; a circumstance analogous to the appearance of miliary vesicles amongst rashes of rubeola." 277.

Thus the venereal roseola is the plane, (we suppose a horizontal one)—the puniceous patch, the gentle ascent—and when we climb the papulæ, we reach the summit of the mountain. As we said before, we think the exanthematous eruptions and the papulæ slide into each other, and we cannot quite agree with Mr. Judd when he declares, that, at first sight, they appear "as widely different and as unconnected as the Zenith and the Nadir."

The order Papulæ is divided by our author into two varieties—Papulæ Elongatæ, and Lichen. The former, he believes, to be a mere enlargement of an originally formed part of the skin, and the latter to be altogether a new production.

1. *Papulæ Elongatæ.*—We hope that the following description will prove clear and intelligible to our readers. We confess ourselves unable to put it into our own language, and lest we should unwittingly misconceive our author's meaning, we offer it in his.

" The papulæ elongatæ appear to be merely the natural rough asperites, or papillæ, as they have been termed, of the cuticle enlarged and distended in wheals during the excitement caused by one kind of venereal virus, (for doubtless the cuticle has vessels that carry albuminous fluid, vide preparation before alluded to in the museum of St. Thomas's Hospital) attended by partial fulness, from turgescence of the cuticle, and inflammation of the parts immediately beneath it; forming a sort of red cutis anserina. These little red projecting bodies, I might mention, can occasionally be traced over the tunica conjunctiva, as in the case of C—— S——d. A few solitary lichen frequently came out about the same time, and have afforded a good opportunity for comparison. This venereal eruption is accompanied by sore throat, and terminates in furfuration, but is a very scarce form of cutaneous affection.

My belief, as to papulæ elongatæ being the produce of venereal inoculation, is most happily strengthened and borne out, not only by the cases given, but by the new method just discovered in France, of causing enlargement of the papulæ by inoculation (*with lymph taken from a scarlatina papule*); at all events proving that the secretion from papules has power to engender crops of enlarged papules." 280.

Two cases are detailed.

In the first, a sore upon the prepuce and corona glandis, occasioning adhesion of them, was followed by enlarged and indurated glands in the groin. Papulæ elongatæ succeeded, with inflammation of the conjunctiva, which was covered with the eruption, and dimness of vision. When the eruption became less florid, solitary lichen was discernible. Finally, the hair fell off. It was necessary to give mercury.

In the second case, a man had connexion on the 1st April, with a woman of the town. In three days afterwards, there was bubo in the right groin, but Mr. J. could find neither sore nor urethral discharge. On the 15th, the glands in the left groin were enlarged. On the 25th, a small ulcer was first visible by the side of the urethra. On the 6th of May, it had surrounded the orifice, and was cupped, foul, and sloughy. On the 12th, the sore had ceased to spread, and by the 23d it was healed. The buboes suppurated. On the 30th, there were developed papulæ elongatæ, with lichen, and diffused redness of the fauces. On the 5th of June, nocturnal pains were added. He was now put on mercury, and gradually all the symptoms passed away.

2. *Lichen Venereus.* Mr. Judd thinks that the average time at which venereal lichen makes its appearance is between the 12th and 14th week after contamination. He thinks also, that "in all probability," it is the produce of a pimple which may, by suppuration of its apex, assume the appearance of a vesicle, pustule, or ulcer. Perhaps much of conjecture, and a little theory break out in the preceding suppositions. Many sores of different characters commence as pimples, and certainly various eruptions follow them.

Mr. Judd has expended much labour and consumed not a little time, in endeavouring to distinguish the exact seat and structure of papulæ. The conclusion at which he seems to have arrived, is, that, during febrile excitement, and increased cutaneous plethora, small portions of coagulable lymph are *deposited* here and there by vessels immediately upon the rete mucosum, but under the cuticula externa, which increase and form lichen; (and thus far vessels carrying red blood have already been traced). The semi-transparent cuticle admits of the red mark caused by these deposits being seen through it; ultimately the lichen advance and project, and a little red *effusion* taking place, stains the rete mucosum, and forms the areola surrounding its base.

This new body or lichen is soon endowed with sufficient vitality to inflame by external friction or constitutional irritation, and becomes capable of vesicating or even suppurating. When the former process takes place, a straw-coloured lymph is given out at its apex, between the coagulated lymph of which the lichen is formed, and the delicate thin cuticle by which it is covered.

He proceeds thus to describe the declension of the eruption. The purest forms of lichen seem to decline by merely losing their vascularity; the over-distended cuticle at the summit throws off a dead scale; and in some cases the small portion of concrete lymph escapes through a little breach in that membrane; the absorbents gradually remove the coagulated lymph that constituted the greater part of its bulk; the rete mucosum forms a new portion of skin; and after a time there only remains a red stain; and before the latter disappears, it becomes white in its centre; and lastly, leaves a pit or depression, that may be both felt and seen in the cutis vera, through the epidermis, marking out the place where the eruption had been situated.

Syphilitic lichen differs from common lichen in the greater disposition on the part of the former, to display large papulæ, containing serous, or sero-purulent fluid, especially where exposed to friction, and on parts covered with hair. Venereal lichen is consequently more prone than the common form to scab and crust.

"In four or five months a second set of lichen not unusually follows the first, (the latter I denominate secondary or deuteropathic lichen,) and if it is of the solitary form, it is almost always of larger character than the primary or first eruption is,—fewer in number, disposed to be vesicular, often contains opaque lymph, forms incrustations, and frequently is blended with favi. The eruption is often set upon the skin generally in the form of triangles in rows of threes or fours, and only a cluster here and there; and lastly, these secondary eruptions decline more slowly, but yet more completely than primary lichen, but they leave the party more subject to pains in the limbs." 293.

Mr. Judd has observed that a person who has previously had lichen solitarius is very apt to have it succeeded by lichen circumscriptus or lichen racemosus. The first eruption of lichen is always ushered in by pyrexia, and comes out quickly; the secondary attacks are slow, and almost unattended with fever.

Syphilitic lichen often runs into pustular forms of eruption, and is not unfrequently followed by genuine ecthyma. In one case related by Mr. Judd, it degenerated into tubercles.

Ten cases are related. We shall glance at one or two. We do not think it necessary to illustrate the general characters of lichen, because surgeons must be pretty familiar with them. We therefore select the cases on account of some peculiarities that they present.

Case. *Sore—Solitary Lichen—Tumor on the Os Frontis—Epilepsy.*

A man, æt. 21, perceived, nine days after connexion, four pimples on the corona glandis; they ulcerated deeply, and caused much destruction of the glans and hæmorrhage. He took much mercury, which did not make his mouth sore. Phymosis and much loss of substance of the glans resulted. He remained well for ten months.

In May 1828, he was attacked with pains in his shoulders and knee, sore throat, and pleurisy; which were relieved by antiphlogistic treatment. In June he had pains in the limbs, swollen feet, and lichen. In July a swelling, containing fluid, formed over one parietal bone. On the 18th he had an epileptic fit. He stated that he once formerly had a similar one, after having been in a violent passion. Some serum and blood were let out of the tumor, after which it gradually subsided. It was subsequently divided to the bone, but this was not found diseased, nor did it exfoliate. Early in August the lichen was desquamating. On the 8th, 25th, and 27th, he had a fit, and afterwards they were repeated. From the latter end of June to the 25th of July he took mercury in one shape or other. In September he was put on the nitrate of silver. In November zinc and tonics were ordered. But though the venereal symptoms subsided the epilepsy did not.

It is, of course, difficult to determine on what the epilepsy in this case depended. One thing, however, is certain—that the case was, in the first instance, mismanaged. When it has been so, it is impossible to predict what strange consequences may ensue in peculiar habits.

Case—*Pustules—Abscess in the Urethra—Lichen Solitarius and Lichen Circumscriptus.*

G. S. had, on the 1st September, 1821, urethritis of an inflammatory character. Three days after connexion, a pustule formed on the dorsum of the penis, attended with slight enlargement of the inguinal glands. Then came a swelled testicle. On the 10th, a few more pustules formed on the under surface of the penis. There was much pain in the urethra on micturition, and, on the 24th, the mucous membrane was seen bulging into it, the body of the penis opposite this point being swollen. On the 25th, a probe, passed into the urethra, indicated that the protrusion fluctuated; it was therefore punctured through the urethra, into which a quantity of pus was discharged. On the 27th, another similar abscess was punctured, and, on the 28th, there was no pain in making water.

It will be remembered that the original sore was stated to have been a pustule on the dorsum of the penis, which caused slight enlargement of the inguinal glands. Afterwards a few more pustules formed a second sore on the under surface of the body of the penis; and shortly a third set formed another sore on the pubes, which were soon covered with thick scabs; and on the latter being detached, they each left ulcers as large as a shilling. In October the sores extended by ulceration, and it was December before they healed. In March, an eruption of lichen gradually came out, and the cicatrix of the primitive sore on the penis re-ulcerated, and formed small pustules once more.

He was now directed to rub in the mercurial ointment. This was in May, 1822. In February, 1823, his ailments were all gone. But they returned in May, when a second eruption made its appearance, with the characters of lichen circumscriptus. The following passage concludes the case.

"June 28th.—The whole of the eruption is desquamating. He has had no other venereal symptom, nor has he lost flesh, or suffered from not having mercury used in the cure of his more early symptoms, though doubtless they were caused by the pustule on the penis, which arose from the venereal contamination." 307.

Mr. Judd justly observes, that mercury was not employed in this case for the early symptoms, and he appears, if we understand the tenor of his reflections, to think that the result is not discreditable to the non-mercurial treatment. What Mr. Judd's idea of suffering from syphilis may be, we cannot take upon us to determine, but undoubtedly it must be of a liberal complexion. To us, the present patient seems to have smarted pretty severely. The primary sores commenced in September, and did not heal until December—upwards of three months. Three months after that, secondary symptoms were developed, and for eleven months more he was not well enough to discontinue regular treatment. He was then reported well; but again, before three months had elapsed, a syphilitic eruption re-appeared. We are plain folks, and we do think that the continuance of syphilitic symptoms for a year and a half, must be looked on as constituting rather a bad case.

The army surgeons, as our readers well know, have been the most active in pursuing the non-mercurial treatment of lues. The temptations to let the disease take its course are considerable in the military hospital, for the patient is much under control, and the surgeon may " do what he likes with his own." We cannot be surprised if the opportunities for experiment have been seized by Mr. Judd, nor can we be astonished when we find in his book that such practice was attended with its usual results, protracted symptoms and uncertain cures. Take, for example, the next case.

CASE.—*Sore and Bubo—various Eruptions—Death.*

N. W. æt. 25, had a " suspicious-looking sore" after connexion, with bubo. He was feverish. His bowels were cleared, and he was ordered—

*Ung. hyd.* ʒj. *omni nocte illinend.*

Two days afterwards, Jan. 3, there was more pyrexia. He was directed to take Dover's powder and antimony; but whether the mercury was continued or otherwise we are not informed. The bubo suppurated, the febrile symptoms gradually disappeared, and on the 14th, one *fortnight* after the commencement of the mercurial course, the sore and bubo had both healed, the former, however, leaving much induration. He seemed otherwise to be well, and went " from under treatment."

Here was a patient with a " suspicious" sore and bubo, subjected to a fortnight's course of mercury, if not to less. The sore left induration behind it. Under such circumstances, the experienced surgeon would naturally anticipate the occurrence of secondary symptoms.

On the 2d of March, the patient was attacked with lichen circumscriptus, pain in the right hip, and pyrexia. Purgatives and salines were ordered.

10th.—The pain in his hip is very violent. A large portion of the eruption is becoming brown and desquamating: but there is here and there a fresh lichen out. The patches represented were situate upon the side of the chest, the circle of vesicles upon the chin, the red maculæ on the cheek, and the solitary lichen upon the body and arms. His tongue is very white, and he is still feverish." 309.

No mention is made of mercury being prescribed, yet it must have been so, for, on the 18th, we are told that the mouth was sore; on the 2d of April, that the action of mercury was gently kept up; and on the 20th, that, being sick and purged, the mercurial ointment was left off.

27th. " He has continued the mercury with the exception of two days, when he was unwell, and took a purge.

28th. He has again become giddy, and is much purged.

Omittr. Ung. Hydrargyri.
Olei Ricini, ℥iij. statim sumendæ." 310.

In the night of the 28th, he sat in his shirt in a cold privy, and next morning he was seized with symptoms of peritoneal inflammation. They made head in spite of the treatment resorted to, and on the 1st of May he died.

*Dissection.* The peritoneum was found highly inflamed, secreting pus and serum in large quantity. The omentum, stomach, and intestines, were glued together by coagulated lymph, and flakes of it were found floating in the fluid contained in the abdomen.

The eruption remained visible, though a little paler, and its bases left well-defined stains of red lymph in the rete mucosum and upon the cutis vera.

We see in the preceding case the disastrous consequences that may follow an inadequate course of mercury in the first instance. The fatal peritonitis was, of course, accidental; the occurrence of the secondary eruption was not. When, after an irregular or imperfect course of mercury, a sore leaves decided induration behind it, we may be tolerably sure that some mischief will ensue.

### III. Vesiculæ Venereæ.

These constitute the third order in Mr. Judd's classification. He thinks that vesicles not unnaturally follow the papulæ, and we agree with him. Vesicles, it is well known, are very apt to run into pustules, the inflammation not being limited to the formation of serum, but occasioning the secretion of a more puriform fluid. This is the case with venereal, as with other vesicular eruptions.

Mr. Judd presents us with no consistent or detailed account of the venereal vesicular eruptions. Having stated that they exist, and offered a few remarks on the anatomical characters of vesicles, he falls back upon his cases, which constitute both text and illustration. This is convenient for an author, for it saves him the trouble of collecting his ideas, and of drawing conclusions from the facts that he has witnessed. But, in proportion as the plan diminishes the author's labour, it augments the reader's. He toils through many cases, perhaps obscurely worded, and, unless he is gifted with the sagacity of a critic and the practical knowledge of an experienced surgeon, he sighs to find how difficult it is to arrive at any satisfactory or definite opinions.

Vesiculæ venereæ, says Mr. Judd, generally appear from two to three months after contamination, but it is almost impossible to make out the precise time required, from our not knowing the exact moment of absorption. The eruptions seem to consist of these varieties; viz. herpes solitarius, confertus, circinatus, rupia, and rupia prominens venerea. This concise account of herpes is illustrated by seven "very select and interesting" cases. Of these seven cases, four are specimens of herpes solitarius—two of herpes confertus —and one of herpes circinatus.—Of the first four cases we shall notice two, because they serve to shew the very different effects that inoculation with the very same poison will produce upon different individuals.

Case.—*Vesicle on the Penis—Ulcerated Sore-Throat—Pains—Eruption of Vesicles.*

R. D. æt. 32, Aug. 1, 1832. He has a circumscribed, round, elevated surface, or red pimple, with a flat summit, on the glans penis; it does not contain any fluid. This raised spot was first observed five days after connexion, and it seems to make very slow progress, if any. He has urethritis, and got the ailments from a prostitute in —— Street, who also gave the disease to the patient whose case will follow. The slightly raised pimple flattened down, and appeared to subside without any breach of skin: but still there remained a crimson stain of about this shape and size—O. It was brownish in the centre, and looked as if it would desquamate.

On the 28th of September, the red stain appeared to remain in statu quo. But the patient did not, for he was feverish, with pains in the shoulder and elbow, and had an ulcerated sore-throat. Salines and an acid gargle were exhibited.

On the 30th, an oval vesicle appeared upon the thigh, and another on the side of the trunk. On Oct. 1st, various parts of the body and limbs displayed oval, slightly elevated red patches, each of which seemed formed of three or four cellules, filled with a brownish lymph. He had several papulæ and incrustations on the hairy scalp.

Papulæ continued to form for some days, lymph being subsequently secreted in their apices. They slowly increased in circumference, and the lymph then dried into transparent brown scales. The bases of other papulæ became sores, on which incrustations formed and spread.* The flattened red round surface that had originally formed upon the penis, now began to throw out a little lymph from beneath the scale that had for some time covered it.

The ulcerations resulting from the eruption appear to have been severe. On the 14th, the pimple on the penis had formed a clean sore.

"19th.—There are three or four other red protuberances, that are still throwing off thin scales and a little serum, and are likely to ulcerate just in the same way as some of the larger ones have done." 327.

About twenty ulcers were thus formed. They gradually cicatrized, and on the 7th of November we are told that they were just well. On the 9th, two days after the sores are said to be just well, the report states that "his health is re-established," and in this way the case terminates.

Before we proceed to the next, we would offer a remark or two on the preceding. We presume that the patient was a soldier. No private patient would consent to let his symptoms run on as they did here, without any mercury being employed. This is a fair instance of what usually results, and of what may be expected from the non-mercurial treatment. Protracted ailments—an uncertain cure, are most commonly its consequences. We do not think that Mr. Judd can fairly report the case as cured, when the ulcerations left by the eruption were barely healed at its nominal termination. We all know how liable secondary eruptions are to relapse, even under favourable circumstances. In the present case, the cure is not worth a farthing.

Case.—*Sore and Bubo.*

Jos. M., Aug. 31, 1832.—" He has a sore that came as a pimple five days after connexion, and he also has a bubo. These ailments were got within twenty-four hours of the same period, and from the same woman that gave disease to Robert D——d: but he neglected them until now.

Pulv. Jalapæ Comp.

Hirudines x. ad ing.

---

* This is the manner in which we would render the passage in the text:—"The incrustations are increasing on the other sores that were the bases of some of the more opaque papulæ." Our author's inexperience in writing produces much confusion of language, which is not corrected by the useful merits of precision of ideas or methodical arrangement.

Lotio Plumbi Acetatis dil.
Inf. Sennæ, c̄ Mag. Sulph.

September 1st.—He has had many stools, and the groin is less inflamed.
6th. The groin feels as if fluid had formed in one of its glands.

Empl. Lyttæ ad inguen.

12th.—The fluid appears to have been absorbed, and the sore on the penis has healed.

15th.—He remains well. This case would not have been deemed worthy of detail, had it not arisen from connexion with the very same woman that caused all the disease in the preceding rare and curious case: but how are we to account for Robert D——d having secondary symptoms? and Joseph M——ge, who really was contaminated from the same source, escaping with sore and bubo only? I conclude he had but a local effect produced by the poison. There is another very curious coincidence exhibited by these cases: *e. g.* M——ge's pimple commenced on the fifth day after connexion, and so did D——d's; the former got gonorrhœa, but the latter escaped; and M——ge had an enlarged gland, whilst D——d had none. These latter circumstances must depend upon some peculiarity, as to absorption, or of constitution in the party inoculated." 329.

The sticklers for the separate existence of various venereal poisons find it difficult to explain such cases as this and the preceding. Why should different forms of sore, and different constitutional contaminations, ensue from the same infection? Nothing, however, is more common. Two men, having connexion with the same woman, will comparatively seldom contract the same form of disease. Our author's cases are rather queer. In the present instance, we are told on the 12th that the sore is healed, and on the 15th that the patient remains well. Now as secondary symptoms do not usually occur in three days after the healing of the primary sore, we presume that the case, so far as it goes, is not conclusive against their appearance. Of course we can only judge of the case as it stands. Our author does not mention his having seen the patient since, and we must, therefore, presume that he has not.

Two cases of "herpes confertus" are detailed. We know not that they possess any other useful qualification, than, by shewing the effects of injudicious treatment to teach us what we ought not to employ. For example:

*Case.* A man, nine days after connexion, found two circular clusters of vesicles upon the penis, which gradually formed sores. His mouth was kept under the influence of five grains of blue-pill nightly for three weeks, when the mercury was discontinued. The sores had healed, but left indurations behind them. Seven weeks after this, an eruption of vesicles appeared upon the body. Then followed sore-throat, pains in the limbs, two other attacks of vesicular eruption, inflammation of one epididymis, violent nocturnal pains. Eight months after the commencement of the disease he is reported well, although the report of the previous month stated that the eruption was not gone.

Thus we see a case drag on for eight months, and then terminate in a dubious way. But observe the circumstances. The primary sores were treated by a very short and inefficient course of mercury, and indurated cicatrices were left. Who can wonder at such a result under such management? Who can be surprised at the idea which prevails, that in the army the treatment of syphilis has become neither one thing nor the other—just enough mercury being given to obscure the symptoms, and to render every indication perplexed. We are no advocates for the non-mercurial treatment of lues, because we have seen and daily see its miserable failures. But we would rather give no mercury at all than give it in such a way as to—" keep the word of promise to the ear," and keep it to the ear only.

In the very next case a sore was left with "extreme induration." The patient went from under treatment apparently well, but returned four months after the commencement of the complaint, with "a set of opaque vesicles almost like ecthyma." He "afterwards finally recovered."

Herpes circinatus is exhibited in a case as illustrative as the preceding of the benefits derivable from the imperfect exhibition or total neglect of mercury.

*Case.* A man had a round thick black scab, covering a sore on the dorsum penis. It commenced three days after a suspicious connexion at the back of the Bank.* Poultice and jalap were the treatment. The scab separated and left a sore to which calamine cerate was applied. Herpetic vesicles now came out on another part of the penis, and in a few days another cluster followed. Three weeks after the commencement of the complaint, we find the primitive sores healing fast, but no other treatment than what we have mentioned is stated to have been employed. In six weeks more roughness and tenderness of the tongue and sore throat appeared—then mottled redness of the skin, and penis—then herpes circinatus. At the end of six months we are informed "his throat is well, nor has his health been much pulled down by this long-continued malady, and he now is in strength and comfort."

Mr. Judd remarks that he purposely abstained from the use of mercury for the eruption, in order that he might be enabled to watch it. But that is no reason why he should abstain from its employment for the primary affection. Rational experiments are very proper. But desultory and arbitrary ones are seldom serviceable. They teach the observer little—establish no general inductions—and entail on their unhappy object, be he man or brute, much unnecessary suffering.

We have before remarked that Mr. Judd, being evidently unaccustomed to writing, labours under the disadvantage of not being always perfectly intelligible. But the obscurity that often clouds his pages cannot always be laid to the fault of language, but is at times attributable to the ideas which it conveys. It is difficult, for example, to determine precisely what our author means, with respect to the nature of primary and secondary herpetic vesicles.

* The following observation of Mr. Judd's is naïve. " I have attended several patients that got contaminated by the same women (their venereal histories are herein detailed), and their primitive sores led to secondary eruptions in every instance, which is rather an unusual circumstance." When we see, as we shall anon, how this case was treated, and conclude, as we suppose we may, that other similar ones were similarly managed, our surprise at the occurrence of secondary symptoms will materially diminish.

"Persons," he remarks, "that long have been subject to gleet, on the discharge ceasing, frequently at these intervals become affected with herpes præputialis, or labialis, at times, and returns of the discharge, or of the eruption, alternate with each other during years ; of the former I have now seen many instances ; one that has lasted a dozen years, and without any sufficiently evident reason. Catching cold seems to be a common exciting cause (though not perhaps the proximate one) of a similarly formed eruption upon the lips. The too long use of the same pocket-handkerchief certainly produces the latter in some; whilst others imagine that they trace the former to deranged bowels. This is almost as frequently met with on the body of the penis, or on the scrotum or thighs, as any where else ; indeed, after its raised base and itching vesicles have once attacked the prepuce, or penis, it as frequently afterwards comes out on the thighs, or buttocks, or other parts, without recurring to the penis also ; and, in a few rare cases, numerous patches appear over the whole body ; such things happen, and, as they are scarce, some such cases have been placed in the following pages." 332.

Herpes præputialis, herpes labialis, sores commencing as vesicles, and vesicular eruptions, are all grouped together, and, for aught that we can determine, are set forth as depending on much the same causes. Yet those causes, though apparently dissimilar, would seem to be regarded with an equal eye by Mr. Judd, who places in the same category intermittent gleet, cold, costiveness, venereal intercourse, and a stale pocket-handkerchief. What practical deduction is to be drawn from these remarks, what deduction is to be drawn from the cases, we confess ourselves quite incapable of guessing. The reader may indeed form *his* conclusion, but probably that will not be the author's. We must hurry on.

### IV. Rupia Venerea.

This forms the subject of five pages of description, and of nine cases spread through nearly forty pages more. To the practical surgeon, as to the speculative inquirer, rupia presents a curious problem. The former is anxious to cure, the latter to understand and to explain it. But rupia frequently baffles them both, and whilst the patient goes on from bad to worse the former expends his remedies, the latter racks his ingenuity in vain. We know not if it was fair to expect from Mr. Judd, a careful consideration of this most mysterious of secondary venereal eruptions. He does little save offer a sketch of the phases of the vesicle, and venture one or two statements on its source which we cannot receive without misgiving.

Mr. Judd divides rupia into two varieties :—1, rupia simplex ; 2, rupia prominens. The following is his description of the former.

"Rupia-simplex-venerea is generally preceded, like the other variety, by deranged health and anorexia. It commences by red patches, scarcely elevated, almost like stigmata, which gradually come out over the limbs and shoulders, seldom over the body. They extend in size, from a pink serum being thrown out into them, but advance so slowly that only a few of the earliest have taken on vesication by the end of the third week after their appearance. The cuticle over the red spots thickens, and becomes marked by white lines, as if about to desquamate, and gradually it becomes elevated, by a thinner secretion taking place under it. So soon as the cuticle covering these parts is thus raised into a large flat, it becomes of a brownish, or even white hue ; for, having lost its transparency, it no longer transmits the reflected crimson colour of its base: and, shortly after this period, the portions of cuticle covering the various vesicles is every where detached, except just at its edge, and may be wiped off. On removing the thick sanies immediately it may be seen that the secretion is thrown out, not generally by one large raw surface of exposed rete-mucosum, but that it issues from two or three smaller irregular surfaces, previously concealed by the elevated portions of cuticula-externa, lately forming the cap of the flat vesicles. The sanious lymph now dries upon the exposed surface, and forms a dark brown, or almost black, thin plate or scab, often at first lying in a pit below the general level of the surrounding skin; the sore still increasing by vesication at its edge, in the course of twenty-four hours its secretion forms another more extensive thin plate, or scab, under the former; and similar processes are repeated, until it may be observed that each crust is evidently formed by successive layers of dried discharge, each of greater extent than that of the preceding day, so as always to keep the increased size of the scab equal to the increasing diameter of the sore, but in this first variety it never becomes so thick as in the second, or rupia-prominens. The bases are not surrounded by very extensive inflammations, or areolæ. About this stage of the disease, soreness and tenderness are experienced and complained of in the ulcers; and the scabs become softer and are easily detached from the secretion being more puriform; and, ultimately, very foul ulcers are left by them to be filled up by granulations." 342.

Every patch of venereal rupia leaves a large crimson stain.

Such is Mr. Judd's description of the simple form of rupia. It is well enough as far as it goes, but it is little more than a mere catalogue of appearances. The circumstances under which the eruption occurs, the progress of the *disorder*, the results, are imperfectly mentioned farther on, and a mere anatomy of a sketch supplies the absence of copious and accurate description. On rupia prominens Mr. Judd scarcely touches, contenting himself with a reference to Bateman's excellent account of it.

Mr. Judd expresses well grounded astonishment at Dr. Bateman's not even hinting that rupia is at times a secondary venereal symptom, nor remarking its concurrence with ulcerations of the throat. The omissions in question are equally singular and great; and it is remarkable how indefinite the opinions of practitioners with respect to rupia appear to be. Those who are not at all acquainted with the characters of lues, usually look upon this eruption as necessarily syphilitic, and lump it with the mass of secondary symptoms. Other surgeons, on the contrary, influenced, probably, by the statements of Bateman, seem to consider rupia merely cachectic, and seldom or never a syphilitic symptom. As usual, the truth lies between extreme opinions. Rupia undoubtedly follows primary symptoms, when mercury has not been used, and occasionally, though very rarely, it is not attended with a broken-down and cachectic state of system. But in the majority of instances rupia occurs, when the constitutional powers are materially impaired, and especially where mercury has been improperly employed.

Its ordinary accompaniments are sloughy ulceration of the throat—nodes —inflammation of the testis—inflammation of the synovial membranes of a passive character—in short, those symptoms which singly or collectively appear in the cachectic patient. Of cachexia it may be deemed one of the most common and most characteristic features—especially of that cachexia which results from the compound operation of lues and of mercury.

Mr. Judd makes a startling announcement. "Rupia," he says, "and rupia prominens venerea are the produce of a primary vesicle upon the penis."

In this statement of a fact, we see clearly enough the cloven foot of theory. Because rupia is vesicular, it is theoretically right that from a vesicle it should be derived. But a little reflection will induce us to pause before we attach much importance to so sweeping a dogma. Many venereal sores, of various descriptions, originate in vesicles. The primitive form of most syphilitic ulcers is uncertain, and a papula or vesicle may constitute the commencement of all. Our information on this head is imperfect. If rupia, then, frequently springs from a vesicle, it probably does no more than many secondary eruptions of opposite character *may* do also. But cachexia may be induced by the operation of cold, intemperance, and mercury, in almost any individual affected with venereal primary symptoms. When cachexia is induced, rupia is usually one of those symptoms, the aggregate of which constitute the cachectic state. Here, then, we see that secondary agents (for example, intemperance and mercury) will induce rupia in cases where the primary sore has been of the most opposite description.

The tubercular eruption is perhaps the most opposed to rupia, not only in its own anatomical characters, but in all concomitant circumstances. If rupia necessarily succeeds a vesicle, it is not likely that the tubercular eruption should do so. Yet we have seen the tubercular eruption rapidly converted into rupia by injudicious depletion, and the coarse employment of mercury. We remember well a remarkable case of this sort. A patient had tubercular eruption, enlargement of the tonsils and yellow ulceration in them, and an indurated cicatrix. He was ordered to rub in large quantities of mercury, and to live low. He had been previously accustomed to taking large quantities of ardent spirits, of which he was suddenly deprived. The consequence was, that the ulceration in the throat deepened, and became a sloughy sore—periosteal inflammation occurred on the inner malleolus of one tibia—and the tubercles first scabbed, then ulcerated, and many of them became converted into what could not be distinguished from rupia prominens.

When we look narrowly at our author's statement, it is either indefinite from its general application, or erroneous from its limited: in either case, it is of little value.

"Genuine rupia (he continues) is at times accompanied with cough, as in puniceous patches and pleuritis; and ulcerated legs and swelled ancles are met with in this disease. Wandering pains in the head of the tibiæ and in the ulnæ are not uncommon in the prominent form. Inflamed synovial membrane at times exists in these cases. Chronic enlargement of both testicles is usual, even in patients who never had urethritis. Occasionally the eruption is preceded or followed by tubercles, and in the worst cases by nodes and corona veneris, with exfoliation from the cranium." 344.

The preceding symptoms are such as mark the cachectic state of system. Patients in that state not unfrequently become affected with ulceration of the mucous membrane of the bowels—some become phthisical—others are suddenly carried off by latent pneumonia or peritonitis, or by erysipelas. In the Lock Hospital, every year brings its five or six victims to cachexia. From what we have seen, we should say that the majority die with symptoms of low continued fever, and present after death congestion of the intestinal mucous membrane, or ulcerations of the clustered glands of Peyer. When the cholera prevailed in the Lock, several patients with cachexia and rupia died of it.

Mr. Judd relates nine cases of rupia. Our space does not permit us to introduce more than one. We select it, because it shews the fatal effects of syphilis on peculiar constitutions.

*Case.* A gentleman, æt. 27, on Thursday in the last week in May, 1819, after dining with some friends, and taking rather free libations of wine, slept with a young woman with whom he had intercourse three times. In the morning he observed (in his own words) "three heads" on the skin of his penis (probably vesicles), and felt a soreness of the prepuce. He imagined them to be a trifling ailment, and applied goulard wash and cold-cream. On the Saturday following he left Town with some friends, still indulging in full living. On his return, the vesicles (or whatever they were) had become sores, and were extending. On the Saturday following, he went to an eminent surgeon, who pronounced the sores to be chancres. Doubts existed in his mind, and the two women he had been with, and known during the preceding three months, underwent a medical examination, and were both found to be free from disease. This was very properly not considered conclusive.

By the 1st of June* the sores had begun to throw off sloughs, and two others had broken out. On the fourth day their appearance improved, but they still continued irritable, and had a disposition to spread. Under the use of black wash, they were healing fast, when, on the 14th, a bubo appeared in the left groin. He was now ordered—

*Ung. Hyd.* ʒj. *o. n. infricand.*

On the 26th, another swelling commenced in the glands of the right groin. The mercury was continued, but his mouth was only a little sore, and scarce an approach to salivation ensued. He had an attack of urticaria, and was then ordered—

*Pilul. Hydr.* gr. iv. *bis die.*

Soon his throat became extremely sore, and, on the 1st of July, the blue-pill was discontinued. Warm sea-water baths at Brighton were resorted to, but they produced excessive lassitude. What the treatment was, it is impossible to determine, for a peculiar confusion distinguishes this, as most of the cases contained in the present volume. It seems, however, that sarsaparilla was given in small quantity.

In the latter end of July, a vesicle appeared on the forehead, and soon ulcerated to the size of half-a-crown; and others came out upon the neck, shoulder, side, and back. The throat continued ulcerated and sloughy. Pains then began in the temples, forehead, and joints, and his weakness was so great, that he experienced a constant lethargic sensation. The unfortunate gentleman now came to town, and placed himself under Mr. Judd's care. He was in a state of great exhaustion—his voice had become nasal and snuffling—and he had patches of rupia—his throat was sloughy and ir-

---

* This is dated in the Report June, 1820, but surely that must be a blunder. —REV.

ritable, with a vast secretion of mucus and saliva—he had enlarged inguinal glands, anorexia, and could scarcely walk. Sarsaparilla—the oxymel of squill gargle—quiet, and country air, were the remedies prescribed.

In September, the throat was healed, the rupia gone, and the health improving daily.

In November, he went out coursing, and got very wet. In a day or two, his throat re-inflamed, and in a week was covered with a large slough. He recovered from this, with the loss of the uvula.

In the following year, he contracted in the country fresh venereal ailments, followed by such severe secondary symptoms, that he did not live the twelvemonth.

There is no doubt that the venereal poison is infinitely more deleterious to some constitutions than to others. Some persons will contract syphilitic sores repeatedly, neglect them entirely, or perhaps be treated injudiciously, and yet will suffer comparatively little. Others, on the contrary, will become affected with the most aggravated symptoms, or even lose their lives, when the utmost care and the ablest possible management have been employed. There is no complaint in which the truth of the old proverb is so palpable :—" One man may steal a horse, when another may not look over a hedge."

On reverting to the remarks of Mr. Judd upon rupia, we must deplore their meagre and hungry character. Had many of the cases that bloat the volume been omitted, and had more general observations and conclusions with respect to venereal symptoms been presented to us, we should have derived more benefit than we are now likely to do from the study of the book. We pass to the next, the fourth, order of eruptions, the—

### Venereal Pustules.

The order of venereal pustules comprises three varieties of ecthyma :—Ecthyma phlyzacium—ecthyma psydracium—and ecthyma magnum.

Mr. Judd, who is always able to discriminate the primary affection giving rise to any secondary eruption, informs us that the venereal ecthyma commences by a primitive small pustule (rarely a suppurating pimple on the penis). As a proof of the correctness of this opinion, he observes that he was so fortunate as to see the primary pustule in several cases. This statement requires a little examination, because the consequences which flow from the position are of more importance than might at first sight be supposed.

Either Mr. Judd intends that his observation should be taken in an unqualified manner, or he does not. If he does not, he should have modified it, and contented himself with remarking that, in some instances, ecthyma is preceded by a primary pustule. If, upon the other hand, he does lay down his position as a general one, he must be sensible that one or two, or even several cases cannot prove it, should any material objections to it exist. We think that there are such objections.

1. In the first place, we have seen lichen follow a primary pustule on the penis. A gentleman came to us with a distinct pustule on the outer prepuce. He was married, but had had connexion with a common prostitute five days previous to the appearance of the sore. On the day succeeding that appearance we saw it. From circumstances that we need not mention, this gentleman employed no mercury, nor did any thing further than take a little opening medicine, and apply spermaceti ointment to the ulcer. In six weeks after the commencement of the sore, he was attacked with true venereal lichen. For this we put him through a course of mercury, and he perfectly recovered. We merely mention this case en passant. It is clear that, if a papular eruption will succeed a primary pustule, the latter is not likely to be the essential antecedent to a pustular eruption.

2. There is no doubt that a person labouring under any form of primary sore, or secondary eruption, may, if brought into a bad state of health, be attacked with rupia or ecthyma. Of this we every day see instances, and, indeed, ecthyma is most commonly witnessed in the mixed cases, those where mercury or some other debilitating agent has broken down the constitution, and given rise to the vesicular and pustular eruptions.

If, then, an eruption, not pustular, will follow a primary pustule: and, if a pustular eruption may be induced upon almost any primary or secondary affection, by certain debilitating agencies, it is most unlikely, indeed it is impossible, that ecthyma should necessarily follow a pustule. No doubt the same character very frequently pervades the primary and secondary symptoms. But, though this may be partly owing to the specific nature of the particular virus, it is principally, we believe, due to the state of the patient. If primary phagedæna is followed by cachectic secondary symptoms, there is little in that to excite surprise, because the individual must, in each instance, have been in a condition adapted for the extension of ulceration and of sloughing—in fact, must have been in a very bad state of health. We know of nothing in practice more important than the recollection of this fact—that the character of the secondary symptoms, and, indeed, of the primary sore, is mainly dependent on the state of the patient's constitution at the time. This is the great point to be recollected in treatment—this is the only safe principle to guide us. If we are led away from attaching importance to it, by theoretical considerations of the particular sore that determines each particular eruption, we are certain that it will be productive of disastrous consequences to our patients.

The resolute tendency to theory on our author's part is sufficiently obvious in the following passage, which follows hard on the position to which we have already directed attention.

"I am well aware that the patient more generally presents a deep round sore to your notice; but that is only the remains, or ulcer, left on the base by the original pustule, and it seldom can be otherwise; for the pustule, being covered by the thin and delicate skin of the prepuce and glans, in most cases is soon broken up by the patient's clothes, even before he is aware he has a sore; and it probably is the sensation from the raw surface, occasioned by the torn skin, that first draws his attention to the part." 385.

When these observations are scrutinized, they plainly amount to this:—In the majority of instances, we can find no pustule; but it must have existed notwithstanding, because—it *ought* to have done so. Let us insert our author's description of ecthyma.

"The fever ushering in ecthyma commonly arises about fourteen weeks after impure connexion has produced the primitive sore. The eruption is preceded by some days of lassitude, especially when it is about to break out, and also by pains in the side of the neck, and about the situation of the levator scapulæ and scaleni muscles; likewise at the points of the shoulders, and in the hips and hams, and over the tibiales antici muscles, and down the flat surfaces of both tibiæ. They are most troublesome in the night; and, not unfrequently, this state is preceded or accompanied by a low chronic redness, or inflammation of the mucous membrane; or even by ash-coloured spots (perhaps of ecthyma) in the throat, by cough without expectoration, and a pain in the side, a little before the eruption appears. Areolæ then form gradually in various number, and of a small size, over the forehead, body, and limbs: the face frequently escapes. The red bases have pus secreted in them, and continue to increase during the first few days, until the skin is covered by pustulæ, almost as thickly as in small-pox.

Ecthyma are seen, at the same moment, in various stages of progress, rising, maturating, incrusting, and scabbing. In the commencement, their bases only are surrounded by an areola, or circle, not very extensive; yet, where the eruption is numerous, the whole skin becomes reddened. As they decline, the rings of inflammation around their bases become of a crimson or plum colour; or incrust, or even ulcerate, especially the larger ones, when declining on the legs. Some fresh pustules keep coming out during several weeks; and, even at this late period, the disease may be seen in all its various stages of progress.

The smallest pustules desquamate; the next in size dry off in brown dry scabs; the larger discharge, and have thick incrustations formed upon them that lead to superficial ulcerations, especially on prominent parts, as about the shoulders, scapulæ, and legs: on the latter, some pustules become confluent, and, late in the disease, form ulcers. In persons with slow circulations, very large portions of skin are frequently seen of a deep crimson or chocolate-brown colour; and when the first eruption of ecthyma is gone, it is not unusual to see almost the entire skin of the leg and shin covered with these frightful dusky red stains, marked by numerous dots, depressions, and loss of substance, even down to the cutis, and its cup filled by yellow, sloughy, cellular membrane, with indented edges, and forming, ultimately, deep ulcers. The head, during the eruption, becomes scurfy, or thickly incrusted under the hair from the lodgment of dry pus with exudation, and the hair's growth is materially interrupted, or it falls off and becomes thin." 387.

It will be observed that, in this description of ecthyma, Mr. Judd adverts to the ulcerations not unfrequently consequent on the eruption. Those ulcerations are severe, their characters peculiar, and an accurate acquaintance with their features, history, and progress most important. So far as we can see, the only account our author gives of them is what we have transcribed, an account which is remarkable for its omissions. We would observe that these ulcerations are by no means exclusively the results of ecthyma, but as often follow the vesicular rupia. In venereal cases, the two eruptions are so evidently allied to one another, both in their causes, in the state of constitution which attends them, in the concurrent affections which wait upon them, and in the local effects which they produce, that it is difficult to dislocate them, except in system. On the same individual, vesicles and pustules may be seen together, they run incessantly into one another, and, as we have already hinted, they constantly produce the same sloughy spreading sort of ulceration.

Mr. Judd enumerates the following as concomitants of ecthyma.

"Inflammations of the tarsi, conjunctivæ and irides are frequent accompanying symptoms. Three cases of the latter affection occurred in eighteen cases of venereal ecthyma, and came on from the twelfth to the twentieth week after connexion, almost always later than the ecthyma; but ulceration of the tarsi and corneæ more frequently occurs about the time of the eruptive diathesis.

Ulceration of the tonsils is liable to come on later than the sore-throat, before spoken of,* and exfoliation of bone from the nose, with discharge of pus and blood.

Suppurations of the submaxillary, cervical, axillary, and other glands, are not unusual from the irritation upon the skin, but perhaps as often from the passage of virus through their absorbent trunks.

Abscesses are apt to form in the calves of the legs, and in one instance they were conjoined with very large boils.

Inflammation of the synovial membrane occurs at times in this eruption; and periostitis, and nodes, often follow ecthyma. Ulceration above the malleoli, and in women about the toes, is not unfrequent about this period, as the larger incrustations separate; and swelling of the ankles, or of one of them, or of the leg, even without ulceration, is very constant: this occurred in three cases out of eighteen." 389.

But the vesicular and pustular eruptions may certainly be considered as, *par excellence*, cachectic; at least rupia and ecthyma may be deemed so. The other cachectic affections accompany them, singly, collectively, or in succession. Phagedænic ulcerations of the mucous membrane, either of the mouth and throat, the conjunctiva, the urethra, or the rectum—inflammation of the fibrous system, and of the bones, of a low character, and tending to ulceration and to suppuration—sloughy abscesses in the cellular tissue—atonic inflammations of the synovial and the serous membranes—such are the features of the cachectic malady, which, under the names of pseudo-syphilis, syphiloid disease, and, indeed, under many other designations, has perplexed the inquiries of the profession, and excited the horror of the public. We cannot compliment Mr. Judd on having thrown any light on its nature, or even on having cast a philosophical survey on its characters. A barren and bald enumeration of facts, some of them imperfect, all of them confused, selected without discrimination, presented without arrangement, and connected without system, can scarcely be considered a scientific, or even a tolerable account of a complicated malady. The remainder of our author's observations may be readily disposed of.

" After the more extensive eruption has declined, very small flat pustules, few in number, generally keep coming out near the papillæ that the hairs of the legs pass through. About this period the health of the patient materially improves, but he is still liable to a secondary eruption, and this is described as the second variety in the classification; they are fewer in number, with psydraceous pustules, and therefore called ecthyma psydracium.

Inflammation of the skin of the alæ nasi, or eyebrows, and enlarged testicle, are occasional accompanying affections; and, curious enough, the latter occurs even in parties that have not had urethritis. In one instance, herein related, intense crimson maculæ followed large ecthyma; and mottled skin, or puniceous patches, at times, though rarely, precedes ecthyma, as in the case of J— B—'s. These very extraordinary and interesting circumstances serve as links, to join this venereal eruption with the rest, although Mr. Carmichael will not allow that it is a true syphilitic production." 389.

---

* " Ulcerated throat appeared in two months after contagion—swelled testicle in five weeks."

Our author appears to us to commit a very serious omission in his account of rupia and ecthyma. Both these eruptions exhibit a remarkable tendency to relapse. A patient will have attack after attack of either. We should say that this is a very characteristic feature of the cachectic eruptions, and when we reflect on the state of constitution in which they are developed, it need not excite our astonishment. It is true that most of the venereal eruptions display this disposition more or less unequivocally. Lichen, for example, frequently comes out in crop after crop—the venereal exanthema does the same—but this is more remarkably the case with the vesicular or pustular eruption, of which it may be said—

———— Micat inter omnes.

The cachectic patient, seeming to recover from an irruption of rupia or ecthyma, followed by extensive ulcerations, finds time after time that the recovery is deceptive, the amendment temporary, and fresh attacks of fever ushering in fresh series of pustules, sores, periostitis, frequently consume many months or years. The surgeon should be well aware of this character of the cachectic eruptions, and the patient should be informed of it too. Unless both are familiar with it, the former will commit the most serious errors in his treatment, and the latter will certainly be discontented with medical advice, and sunk in despair with regard to his complaint. In practice, we have many opportunities of observing the disastrous effects of an imperfect acquaintance with this law of the venereal cachexia. Before we quit a subject on which so much is to be said, we will advert to only one other point. Enlarged testicle, says Mr. Judd, occasionally occurs in combination with rupia, and that in patients who have not had urethritis. We have said already, that a general survey of the symptoms of cachexia enables us to arrange them in groups, relating to the tissues they invade. The testis and the mamma are composed in a great measure of filamentous tissue; in both, we have also a mucous membrane; and, added to these, is the glandular or secreting structure of the organs, the exact nature of which is little understood.

The testis is liable to become inflamed in gonorrhœa, because the inflammation may run along a continuous mucous membrane; and we usually find that, when the testicle has swollen, the urethral discharge for a time diminishes or stops. We do not say that the inflammation, when it arrives at the testicle, is limited to the membrane that conducted it. It spreads to the fibrous tissue of the cord, the epididymis, and testis, and sometimes it invades also the serous membrane, and produces in it effusion of serum, of lymph, or of pus. Nay, the external cellular tissue is at times affected also, and diffuse inflammation or abscess in it is set up. But, if observation proves any thing, it proves that inflammation of the testis, in the case of gonorrhœa, is the result of the extension of the inflammatory action along the continuous mucous membrane.

The testis is also liable to become inflamed in cachectic cases, or indeed in company with venereal secondary symptoms not cachectic. The quantity of fibrous tissue and of mucous membrane contained in it are fully adequate to account for the phenomenon. If the sclerotica and iris, the periosteum and bone inflame, why should not the testis? It is in company with one or more of these, that in such cases it usually does inflame, and the surgeon has occasionally the opportunity of remarking successive or concomitant affections of them all. Why it should be so, we need not stop to inquire. That is an ultimate fact. But we may be permitted to observe that it is only by these general inductions, by these wide classifications of phenomena, that we can hope to arrive at any thing like a philosophical acquaintance with the laws of the venereal disease. This is so modified by disturbing agencies, especially by constitutional peculiarities and by remedies, that we fear it must be looked on as Utopian to expect that we shall ever be thoroughly conversant with its infinitely varied forms.

Passing over some cases, for which we have not space, and which indeed present little to arrest attention, we proceed to a chapter, dedicated to the consideration of—

### THE MORE INVETERATE SECONDARY SYMPTOMS.

To do so, we must disregard for the present, the 5th, 6th, and 7th orders of venereal eruptions—the maculæ—the tubercles—and squamæ. Our reason for this course is simply this. Rupia and ecthyma are themselves the leading features of that cachectic state, in which the "inveterate secondary symptoms" occur. It is more natural then to associate than to dislocate them, and we shall reverse Mr. Judd's mode of proceeding.

Mr. Judd can seldom avoid the opportunity for speculation. His short remarks on urethritis contain almost as many theories as lines, and his venereal eruption is generally ushered in by an unpretending assumption, that such or such a syphilitic sore is the veritable Amphitryon, the true fons et origo of the eruption in question.

The practical surgeon who daily sees nodes, ulcerations of the soft parts, and caries or necrosis of the hard, in connexion with rupia and ecthyma, and after almost any primary symptom, provided that the constitution has been broken down, will be inclined to smile when he finds that Mr. Judd attributes even these affections to a special virus.

"The effects," he says, "of a variety of sore slightly spoken of in the last chapter yet remain to be described; it is followed by bubo, and in two or three months by periostitis, nodes on the os frontis, ulna, or tibia, as may be seen by referring to the Cases of J——n and T——es; but no cutaneous eruption, as far as I have been able to make out, is ever produced by the virus of this, or the preceding kind of sore mentioned at p. 483. Having already given examples of the venereal eruptions, both in their earliest and later forms, I shall now conclude my description of the effects of the syphilitic virus, by detailing more obstinate cases wherein eruptions scarce occur, or only form occasionally, and then sparingly, and as one of the latest of this class of secondary symptoms.

The disease in these instances probably is produced by a more intractable form of virus, causing the very worst, most obstinate, and long-continued train of venereal sufferings, that the profession are called upon to treat.

The virus above alluded to appears to attack the nares, the palate, the tonsils, the throat, and synovial membranes, amongst the soft parts; and the periosteum, the cartilaginous and osseous structures amongst the hard ones; wearing the system by nocturnal pains, consequent restlessness at night, conjoined with debility and hectic fever. These symptoms appear slowly, and months, or even years, after this particular form of virus has entered the system, by producing a small superficial sore, which probably should be deemed the only true chancre." 489.

We confess we are in amaze. The world has been engaged for the last half-century in hunting for the true chancre, and has found it as difficult to obtain as the basilisk that Zadig met the damsels searching for. John Hunter picked out one sore, and the "Hunterian chancre" was deemed the archetype of lues. Lately, a very speculative gentleman, Mr. Wallace, of Dublin, has patronized another sore, and advocated its claims to the distinction. But Mr. Judd outdoes his predecessors, for he selects symptoms that have been ever deemed apocryphal, as the criteria of his genuine pox. We have read and read again the passage we have quoted. We doubted, if some hidden meaning was not couched under the apparently obvious sense, for no one who peruses our author's work will in any place feel confident that he understands him. But no; we perceive no reason for suspecting that he means or can mean any thing but this :—Diseases of the bone, and those that he classes with them, are the consequences of a small superficial sore, and must be deemed as the evidence and the effects of the only true and orthodox chancre. If we thought that this opinion was really likely to obtain assent, or even to seem plausible in the eyes of the profession, we should set about exposing the gross fallacies that it embraces. We would show that the most "inveterate symptoms" follow and accompany rupia and ecthyma, and that these, as well as they, are plainly more connected with the states of constitution than with any special primary virus. But we do not think it necessary to enter into a formal communication of such an olla podrida of doubtful doctrine and tangled facts as the following quotation will serve to exhibit.

"These peculiar cases are usually marked out by pains experienced on each side of the head, as after poisons taken up from wounds in the hands; or as in exanthemata and other disturbances to the brain through the system; pains in the neck, shoulders, chest, and limbs, followed by ulcerations of the tonsils, palate, velum, and nares; shortness of breath, cough, and expectoration, as in rubeola; loss of sleep and flesh, debility, swelling and ulceration of portions of the skin of the face, and the ala nasi; incrustations on the brow, nose, and roots of the hair; pustules, boils, tubercles or ulcers, diseased testicles, destruction of the vomer, exfoliation of the ossa palati, pain, and stiffness of the joints, with inflammation, and effusion into their synovial membranes; aching in the shafts of the bones, thickened periosteum, perichondrium, and nodes; enlargement of the spinous processes of the vertebræ, of the ends of the sternum, clavicle, ulna, and shaft of the tibia. 490.

It would puzzle even that first of artists, Mr. Robins, to create order out of the confusion of this extraordinary catalogue. But difficulty crowds on difficulty. We thought that this grisly family of ills arose from "a small superficial sore." We gave the passage which seemed to say that, but we stated that we entertained considerable diffidence with respect to our unriddling any sentence in the work. That diffidence was not misplaced, for Mr. Judd continues :—

"The secondary symptoms taken *en masse* are so various in their forms and appearances, as not always to follow in the same order or succession, and no patient suffering all of them: they vary, moreover, in each, and seem to create an almost unintelligible chaos, difficult to treat, and concerning which it is still more difficult to know which should be described first. I may state that these venereal secondary symptoms are generally, though not necessarily, preceded by a sore: frequently they are produced by one that has healed in three days; by sores whose cure has required three months; by sores attended by induration, and by others unattended by any; by sores that have been followed by buboes, and by those that induced none;—all, all these varied stages appear in their turn to cause this wretched, and often intractable, train of evils; and to the account of these tertiary venereal symptoms, this division of the Work has been allotted." 491.

After all, then, there need not be a sore at all—or if there is one, it may heal in three days—or it may take three months to do so—or there may be induration—or there may be none. All this is consistent with our own experience, but how it is consistent with the previous statement, we profess ourselves quite unable to determine. We shall therefore say no more about it.

It might be thought by those who have not perused the present work, or this review (it cannot be an analysis), with care, that after the declaration of the "unintelligible chaos" which these symptoms form, a careful and an accurate sketch of them would be presented. But it is not so. The description reflects the chaos that it represents, and the subject remains as unintelligible as before. The great and characteristic features of the malady, are smothered under paragraphs of trivial details, and our author, instead of tracing the broad characters of an ulcer of the nares or the throat, pursues the wandering pains that spring from it, or those natural effects that every body knows *must* happen. It is painful to blame, where it is impossible to praise: and useless to proceed, when our advance leads us only to fresh objects of criticism or of doubt. We must therefore, for the present, allow the inveterate symptoms to continue so, and retreat to our old position before the fifth order of venereal eruptions, the—

### MACULÆ VENEREÆ.

The order comprises three varieties. 1. Spili Coccinei; 2. Spili Cruentati; 3. Spili Cuprei.

1 and 2. Spili Coccinei and Cruentati, are mostly preceded by febrile excitement, but do not seem to appear earlier than twelve weeks after the primitive affection, which Mr. J. believes to be a small abraded surface on the penis. Of course this supposition must be taken for what it is worth. These maculæ, he continues, are of a decided crimson colour, and the edge of each regularly defined. Their figure is oviform and constant,—varying in size from a pin's head to that of a Windsor bean, slightly raised above the surrounding level, and containing red lymph. In the most intense varieties of spili cruentati, almost pure blood is extravasated from the extremities of the cutaneous vessels, under the cuticle, into the absorbent rete mucosum, in which it spreads like fluid dropped on blotting-paper, or like rain fallen on dry flags. They are commonly numerous, formed in clusters, of irregular numbers, as threes and fives, with a solitary one occasionally; the surrounding skin being in the onset several shades redder than in health.

The very handsome painted-like appearance of the eruption remains bright about a week; it then gradually declines, each crimson macula gradually becoming copper-coloured, and somewhat diminished in circumference. As they recede, the stains in the rete mucosum become yellowish in colour, and much resemble fading purpura: in about a fortnight the cuticle generally begins to recover its natural hue; and a little prior to that period, a slight depression is generally evident, whilst puniceous patches, on the contrary, leave a considerable rise. Some portions of cuticle, over the seat of the larger spots, desquamate, and at the end of three weeks all traces of the eruption have usually disappeared. As far as Mr. J. has seen, this affection is unattended by sore-throat, and is only occasionally followed by other syphilitic affections.

A very short mercurial course, he states, appears to promote the absorption of the stains, but none is absolutely necessary.

3. Spili cuprei are of a yellow colour from their commencement. They appear slowly and singly, are very long in formation, more slightly raised, and frequently becoming confluent, form copper-coloured patches of the magnitude of the palm of the hand. They last for years, and are little influenced by treatment. They answer, in short, to pityriasis, and we think it very doubtful whether they can be considered essentially venereal at all.

### Venereal Tubercles.

These constitute the sixth order. They are distinguished according to their configurations, and divided by our author into three varieties, which he names:—1, Phymatosis ovata; 2, Phymatosis annulata; 3, Phymatosis verrucosa.

The general characteristics of the syphilitic tubercles are, according to our author, that they consist of considerably raised and inflamed masses, of a crimson colour—that they are permanent during weeks or months—at times suppurate, though very slowly, or subside very gradually—and that in venereal affections, they are not merely limited to the face, as in Sycosis, which they most resemble in appearance and texture, but not always in shape. We would remark, by the way, that if we limit venereal tubercles to such as will come strictly within the boundaries of this description, we shall exclude venereal eruptions, very frequent, very important, and inadmissible into any other order. We shall see anon that Mr. Judd's definition of tubercle is too strait-laced.

1. *Phymatosis Ovata.* The following is Mr. J.'s description of this, the first and chief of his varieties.

"Phymatosis ovata is an eruption of highly raised oviform bodies, of a crimson colour, about the size of split horse-beans,* generally appearing about five months after venereal contamination, and coming out for days in succession, thickly set over the person, the largest being commonly situated upon the back and the shoulders. Each tubercle appears to be the produce of a low but long-continued degree of inflammation, and consists of a deposition of red gelatinous albumen, brownish at its thin outer edge, but redder, denser, and thicker, towards its interior and base, evidently given out by secretion from the rete vasculosum, and situated in a depression, covered by a thickened and distended epidermis, with its papillæ enlarged.

---

* "I have dissected them, and raised off their cuticle by blisters, etc."

A few days after the tubercles have appeared, the cuticle covering the biggest here and there becomes so distended, by the deposition beneath, as to have its natural creases rased out, and to form a plain surface.

The blood during the eruption is always found inflamed, and after these tubercular semispheres have remained crimson and projecting about a month, they become brown. The cuticle covering them loses its tension, and falls back into its natural lines—still later the cuticle over them gradually assumes a copper colour, as it does in all eruptions containing red lymph; the epidermis then falls into sulci, and the tubercles decline, leaving about the end of the second month flat copper-coloured patches, almost level with the surrounding skin. They ultimately lose their peculiar tint; and the cutaneous papillæ, that were situated over the tubercles, having become dark brown dead dots, by the end of the third month fall off with large exfoliations, leaving broad depressions.

This most highly raised and scarce form of all the venereal eruptions is often accompanied by iritis, by cynanche, and nocturnal pains; its cure is much accelerated by mercury." 437.

Mr. Judd, it will be noticed, affirms that this is the most scarce form of all venereal eruptions, and we are disposed to agree with him. He has known it appear as a primitive form, and he has known it also occasionally follow lichen, ecthyma, rupia, or puniceous eruption.

2. *Phymatosis Annulata.* This also, according to Mr. Judd, is a very scarce form of venereal eruption. The tubercles arise almost like extremely large long and oval crimson lichen: some few of them solitary, others are often grouped in circles, then forming a perfect resemblance to the shape of a very thick raised annulus. This variety of tubercle is also evidently distended by lymph, and is very durable, suppurating slowly, and generally lasting nearly two years. No remedy that he has tried appears to exert any influence over it.

3. *Phymatosis Verrucosa,* comprises venereal warts and condylomata, in our author's description of which there is nothing to detain us.

This concludes, therefore, his account of the venereal tubercle, and we must repeat, what we have already stated, that it is too narrow and exclusive. Such an eruption as the following is extremely common, almost as common, we should say, in private practice, and indeed in hospitals, as any venereal eruption whatever. From six weeks to three months after the reception of the primary sore, an attack of pyrexia ushers in the eruption. Ulceration of the throat, generally yellowish and deep, with thickened borders, precedes, accompanies, or follows it; usually *this* appears before the eruption and attends it.

The eruption itself consists of distinct depositions on the superficies of the cutis, of the size of peas, distinctly elevated, and feeling, when pinched up between the finger and the thumb, like a flattened pea. Their colour is a dull, a brownish, or a purplish, very seldom a vivid red. There is no vesication, no tendency to suppuration, no scab. Usually they are distinct, scattered over the body, and most numerous on the back, the face, the thighs. On the lower extremities, they are always larger than they are upon the upper. Sometimes they are confluent. The eruption slowly runs its course. Gradually, the redness of the tubercles subsides into a duller and more coppery tint—the tubercles themselves become more flattened—and they finally desquamate. If no mercury is employed, there remains for a long time a dullish red stain of the skin, some deposition in it, and a tendency to scale on it. But this state of things seldom lasts long, for fresh eruptions, or repetitions of the old succeed.

If, when this eruption is present, the patient's health should by any means be materially broken, it may gradually be converted into rupia or ecthyma, or become complicated with one or both. Most frequently another change occurs. The old tubercles gradually die off, and are superseded by fresh ones of a different character. These new cachectic tubercles* are larger, flatter, softer, and more confluent than their predecessors. They communicate to the fingers a peculiar doughy or semi-elastic feeling—they occur in masses, from the size of a fourpenny piece to an area of some inches in diameter—they partially *scale*, but more frequently *scab* upon the surface—and they readily suppurate, or run into foul ulcerations.

This is a mere sketch of two forms of a frequent and important eruption. What is it? It is not papular, nor vesicular, nor pustular, nor exanthematic, nor scaly. It is clearly and obviously tubercular. Mr. Judd gives no description of it, presents no niche for it, and we cannot but look upon this as a very serious omission. There is this, indeed, to be said in its extenuation, that different persons not only observe, but narrate what they have observed so differently, that it is possible some of the descriptions we have copied may contain an account of this very eruption. So far, however, as we can read the characters of the phymatosis ovata, they do not tally with those of the simple tubercular eruption we have spoken of, and the phymatosis annulata is obviously another thing.

### Squamæ Venereæ.

These compose the seventh order, and are made up of—1, Lepra Venerea; 2, Psoriasis Venerea; 3, Psoriasis Palmaria Venerea.

Mr. Judd's account of the lepra venerea requires no notice whatever. It is despatched by him in a few lines, and numerous authors present a full account of it.

2. *Psoriasis Venerea.* "This (says Mr. Judd) is the produce of a vesicle, and several months elapse before it is followed by its secondary vesicular psoriasis; and hence, from the extreme length of time that transpires between the infection and the period when psoriasis venerea appears, it generally happens that both surgeon and patient have forgot the date and features of the early sore.

This is a rare form of primitive disease as a venereal product in England; for amongst all the multitudinous eruptions described in this volume, there appear to have been but four cases recorded of this disease.

It begins by excitement in the cutis and rete vasculosum, forming inflamed red surfaces, occasioning a succession of minute quantities of thin serum to be thrown out and deposited in patches and flakes upon the under surface of the cuticle, producing thickening. These portions of cuticle soon lose their transparency and elasticity,—become red, then opaque, die, and are thrown off in

* We say cachectic, because they occur in the state of cachexia.

irregular white and brownish scales from the epidermis of the body. If this diseased action chance to be carried on in a dry part, it forms long fissures,—if in a moist one, deep sores of the same shape, or even ulcerations, as about the anus. In the scalp, eyebrows, whiskers, etc., the exuded lymph collects under the successive layers of scales, and then produces incrustations almost similar to some forms of tinea, and especially to porrigo larvalis." 461.

We have evinced throughout this article considerable surprise at the decided tone in which Mr. Judd points out the species of primary sore, that leads to this or that particular eruption. He seldom evinces any hesitation in deciding, on slight evidence, what is confessedly a ticklish matter. If any thing could point out in a stronger manner than another the hollowness of these positive assertions, it would be the enunciation of the dogma that psoriasis is the produce of a vesicle. Psoriasis is a common syphilitic eruption in women. In them we have an opportunity on a liberal scale, of determining the usual primary affection. That affection is about the most opposed to a vesicle that can be conceived—it is the venereal condyloma. If a woman presents herself with *it*, we may be pretty certain that, supposing a fair length of time to have elapsed, inspection of the throat will discover a white ulceration, and examination of the skin will display psoriasis. These two are, par excellence, the consequences of condyloma. If any fact in lues is certain, this is so. Yet, in spite of this, we find that Mr. Judd, without any sort of reservation whatever, sets down a vesicle as the primary cause of psoriasis.

Psoriasis, he says, is very rare. At the Lock Hospital it is very common. The solution of this discrepancy appears to be—that Mr. Judd has chiefly studied the disease in men, while, in order to obtain correct notions of it, it is necessary to see it on a large scale in both sexes. Psoriasis is not a common eruption in the male; it is in the female that we generally see it. The female is very liable to condyloma, and condyloma is the most common antecedent of psoriasis.

The chapter on the scaly syphilitic eruptions must be deemed peculiarly defective. The ordinary tubercle is not described—its changes and its phases are apparently unnoticed—the peculiarities of psoriasis are unnoticed too—and the laws of the scaly syphilitic eruptions generally are not hinted at. If, on previous occasions, we have lamented the absence of those broad views and wide inductions, which can alone simplify the study of syphilis, our advance has not tended to diminish our regret, but has shewn still more forcibly that Mr. Judd has yet very much to do, before he can obtain the acquiescence of those who are practically conversant with the phenomena of lues, or merit the praise (no mean one neither) of throwing light on the laws of this perplexing malady.

3. *Psoriasis Palmaria* is ranked by Mr. Judd as a variety of the eruption, and a special consequence of lues. It does not very unfrequently occur, but, so far as we have seen, it is always preceded, or accompanied by psoriasis, or other eruptions elsewhere, and does not merit particular notice. We have a gentleman affected with it under our care at this moment. He has superficial ulceration of the tonsils, and posterior palatine arch, stains, inclined to desquamate here and there upon the surface, small scabs amongst the hairs upon the scalp, and condyloma. It is always a troublesome affection. Another gentleman with psoriasis palmaria, has at intervals small white superficial ulcerations of the lips, and tongue, and palate. Both have for some time laboured under the affection.

### Comparative Frequency of the Eruptions.

This is a subject of some little interest. There is reason to believe that in different countries, and under different circumstances of climate, diet, and modes of life, the frequency of particular eruptions varies, and that nothing like a fixed ratio exists. The following is the estimate of Mr. Judd.

"Of all the cutaneous affections, *true* venereal exanthismus, or *mottled skin*, seems most rarely to exist; for not more than two cases were detected after two hundred and fifty primitive sores: but it really is a slight and delicate form of rash, frequently overlooked, except, indeed, when it is daily sought after about the proper period.* What by some has commonly been termed venereal mottled skin, on minute inspection with a compound lens, magnifying fifty times, is not always found to be true exanthema, or rash, but a risen form of eruption somewhat resembling it; and this latter species is fully described under the term Puniceous Patch.

*Puniceous patch and its varieties*, or raised red patches on the skin, are of late years more prevalent: they have been observed to form almost one-third of the venereal eruptions.

*Lichen*, and enlarged papillæ, may be said to be a tolerably common form of syphilitic eruption, as they constituted more than one-third; and we may safely calculate upon every fourth case exhibiting some of the varieties of this Order, either simple, patched, grouped, or circumscribed.

*Taking the varieties* of vesicular eruptions into account, they will be found to be almost as common as the pustular ones; and that the former are of frequent occurrence is shown, by one-fifth of the cutaneous cases presenting various forms of herpes and rupia. It may here be remarked that venereal rupia prominens is rare, and that herpes circinatus venereus is so excessively scarce as hardly to be met with in England, though it is seen, at times, both in Ireland and in India.

*Ecthyma* is a very common form of venereal cutaneous affection, for somewhat above a third of all the eruptions that follow primitive sores is of this denomination.

*Spili coccinei* are rare, but oftener met with than genuine tubercles; and when the former eruption appears, it often constitutes the only cutaneous attack that takes place during the train of symptoms arising from that contamination.

*Tubercula* seem very scarce, as but four instances were met with amongst all these venereal cases: and in searching through a hundred others, I found but one or two instances of well-marked tubercles had occurred; though there were cases of what is called Tubercle by some practitioners.

*Squamous affections* of the cuticle are not an over-frequent product of syphilitic virus; for out of this large number of cases there were but seven of psoriasis and lepra, although they were watched, and from two to six years were permitted to elapse, to give time for other forms of eruptions to degenerate into them;—some of the above were so produced, but occasionally they appear as the primitive eruption following the sore." 474.

The only point on which we differ from our author, respects the tuber-

---

* I generally watch for it whilst applying rollers to buboes, some weeks after the sore.

cular and scaly eruptions. We believe that both are far more frequent than he considers them to be. We have also hinted in a cursory manner an explanation of the circumstance.

We have observed a material difference in the comparative frequency of particular eruptions in hospital and private practice. In the former, we see much of rupia and ecthyma. In the latter, we find chiefly the papular, tubercular, and scaly eruptions. The reason is not doubtful. The patients of Lock Hospitals are filthy, poor, intemperate, and have been (medically speaking) ill treated. The higher classes are more careful of themselves when ill, are surrounded with comforts or with luxuries, and have, in general, good medical advice. It is comparatively seldom that we see a private patient with rupia or ecthyma; we might almost say never, except in consultation, or in the instance of some unhappy wretch, who has gone the round of the London surgeons.

There are some small portions of the work before us which have yet remained unnoticed. Two or three pages are appropriated to an enumeration of the ulcerations of the throat—a few more to experiments on the non-mercurial treatment of syphilis—and a brief chapter is consumed in anticipations of benefits to accrue from the use of the hydriodate of potass. For a notice of these matters, we shall find opportunities and space in our succeeding Number.

For the present, we part from the volume and its author. We have concluded the consideration of the primary symptoms and the secondary eruptions of lues. We have differed widely from our author, and we have differed under the conscientious conviction of necessity. If we are wrong, we do our best to ascertain the right, and what we believe to be the right we say. The critic's task is not a pleasant one. Accident places him in the position of a judge, when inclination and many other considerations would lead him to be silent. But, being what he is, it were cowardice to shrink from the responsibility of his situation, and, so that he has one steady aim, the pursuit of philosophic truth, he may expect and will support much obloquy and some misrepresentation.

We have made it a point to direct attention, at convenient opportunities, to the subject of the venereal disease. It is a subject of great importance, because the complaint is so common that all are called upon to treat it, and so complicated, that very few are even tolerably well acquainted with it. Every practitioner supposes that it is easy to treat a clap. But when enlarged lacunæ, or abscess of the penis, or chronic inflammation of the corpus spongiosum, supervene, too many are confounded with symptoms that they do not rightly comprehend, and either betray a discreditable ignorance, or perversely and injuriously blunder on. If this is the case with the simpler affections, it is still more so with those peculiar symptoms, not unfrequently developed in the progress of lues. How few surgeons or physicians would feel or exhibit a decent confidence in the management of the serpiginous phagedænic sores. We are taking, some one may possibly say, a rare and extreme case. Such cases are neither rare nor extreme; but, granting that they were so, the numerous examples of rupia and ecthyma, of sloughing throats and of diseases of the bones, are mementos of unskilful management somewhere and at some time, more unequivocal and more deplorable than any that declamation could dress up.

In short we do not hesitate to say, that exact and scientific notions with respect to the venereal disease are far from generally diffused in the profession; and we are sure that an attempt to disseminate an accurate acquaintance with its forms, and judicious principles of treatment, will be received with favour and encouragement. On this account, we have devoted much space to the consideration of lues; and, as occasions offer, we shall continue to recur to it.

